# Dual complementary liposomes inhibit triple-negative breast tumor progression and metastasis

**DOI:** 10.1126/sciadv.aav5010

**Published:** 2019-03-20

**Authors:** Peng Guo, Jiang Yang, Daxing Liu, Lan Huang, Gillian Fell, Jing Huang, Marsha A. Moses, Debra T. Auguste

**Affiliations:** 1 Vascular Biology Program, Boston Children’s Hospital, 300 Longwood Avenue, Boston, MA 02115, USA.; 2 Department of Surgery, Harvard Medical School and Boston Children’s Hospital, 300 Longwood Avenue, Boston, MA 02115, USA.; 3 Department of Biomedical Engineering, The City College of New York, 160 Convent Avenue, New York, NY 10031, USA.

## Abstract

Distinguishing malignant cells from non-neoplastic ones is a major challenge in triple-negative breast cancer (TNBC) treatment. Here, we developed a complementary targeting strategy that uses precisely matched, multivalent ligand-receptor interactions to recognize and target TNBC tumors at the primary site and metastatic lesions. We screened a panel of cancer cell surface markers and identified intercellular adhesion molecule–1 (ICAM1) and epithelial growth factor receptor (EGFR) as optimal candidates for TNBC complementary targeting. We engineered a dual complementary liposome (DCL) that precisely complements the molecular ratio and organization of ICAM1 and EGFR specific to TNBC cell surfaces. Our in vitro mechanistic studies demonstrated that DCLs, compared to single-targeting liposomes, exhibited increased binding, enhanced internalization, and decreased receptor signaling. DCLs consistently exhibited substantially increased tumor targeting activity and antitumor efficacy in orthotopic and lung metastasis models, indicating that DCLs are a platform technology for the design of personalized nanomedicines for TNBC.

## Teaser

Dual complementary liposomes inhibit proliferation and metastasis in triple-negative breast cancer.

## INTRODUCTION

Triple-negative breast cancer (TNBC) is a heterogeneous disease, defined by the lack of estrogen receptor, progesterone receptor, and human epidermal growth factor receptor type 2. TNBC, which represents 15 to 20% of all breast cancers, occurs more frequently in women under 50 years of age, in African American women, and in individuals carrying a breast cancer early onset 1 (BRCA1) gene mutation (*1*, *2*). Because of the lack of therapeutic targets and limited treatment options, the prognosis for patients with TNBC remains the poorest among all patients with breast cancer (*2*–*4*).

Nanotherapeutics were developed to improve the safety and efficacy of antitumor drugs, which bring measurable clinical benefits to the treatment of several metastatic cancers (*5*–*11*). However, none of the clinically used nanotherapeutics (e.g., Onivyde and Abraxane) are tumor specific. These drugs depend solely on the enhanced permeability and retention effect to enter the tumor, which can be severely hindered by tumor complexity and heterogeneity (*12*). To overcome this obstacle, “next-generation” nanotherapeutics (e.g., MM302) use tumor-targeting ligands to improve their tumor accumulation. Unfortunately, these therapeutics failed to meet therapeutic expectations in clinical trials due to their limited targeting activity and “off-target” effects (*13*). Recent extensive studies of extracellular vesicles (e.g., exosomes) have shed light on the biomechanisms of naturally occurring drug delivery nanocarriers (*14*–*16*). For instance, tumor-derived exosomes use multivalent ligand-receptor interactions between vesicles and targeted cells to mediate intercellular communication and efficiently deliver secreted proteins, mRNAs, and DNAs (*15*, *17*, *18*). Cells use a complex array of molecular interactions to deliver molecules that, in turn, govern cell functions. Multivalent targeting has been successfully achieved in nanomedicines by using two or more targeting ligands and effectively enhances the delivery of nanomedicines to cancer cells in vitro and in vivo (*19*–*28*). A recent study has extended this strategy to simultaneously target tumor cells and their microenvironment, which significantly improved the antitumor activity of checkpoint blockade immunotherapy (*21*).

We have developed a complementary targeting strategy that imparts precisely matched, multivalent ligand-receptor interactions to efficiently enhance the retention of nanomedicines at targeted TNBC tumors and metastases. Unlike conventional targeted drug delivery systems that present a single ligand, we functionalized the surface of a liposome to precisely complement the molecular ratio and organization of multiple cancer receptors overexpressed on TNBC cell membranes. We hypothesized that this precisely matched, multivalent ligand-receptor interaction between complementary targeting drug delivery systems and TNBC cells would increase cellular adhesion and accumulation at TNBC tumors and metastases in vivo, which, in turn, would improve the therapeutic efficacy of nanotherapeutics.

To test our hypothesis, we first developed an unbiased and quantitative screening approach to select optimal targets for complementary targeting. On the basis of the screening data, we then engineered a proof-of-principle, dual complementary liposome (DCL) composed of antibodies against intercellular adhesion molecule–1 (ICAM1) and epithelial growth factor receptor (EGFR) (*29*, *30*), which are molecular targets of U.S. Food and Drug Administration (FDA)–approved drugs, and liposomal doxorubicin, a clinically used breast cancer nanotherapeutic (*31*). Our in vitro mechanistic studies further revealed that DCLs exhibited three major advantages over conventional “single” and “dual-targeting” liposomes: (i) Cellular binding was significantly increased via precisely matched, multivalent ligand-receptor interactions, (ii) internalization was enhanced via cooperative endocytosis pathways, and (iii) therapeutic efficacy was improved via simultaneous blockade of ICAM1 and EGFR pathways. Last, using in vivo orthotopic tumor and lung metastasis models, we demonstrated that potent tumor-targeting and antitumor activities of DCLs can be effectively translated into therapeutic and survival benefits by inhibiting TNBC tumor progression and metastasis. Together, these data demonstrate that complementary targeting is a promising and translational platform for the design of tumor-targeting nanomedicines.

## RESULTS AND DISCUSSION

### Selection of TNBC targets for complementary targeting

**Fig. 1:**
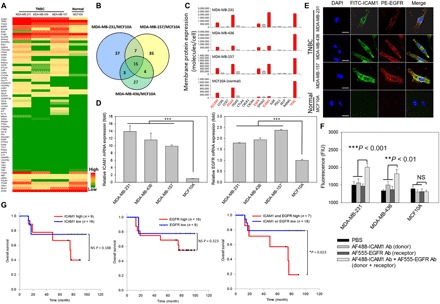
Identification of ICAM1 and EGFR as candidates for TNBC complementary targeting. (**A**) Surface protein expression of 68 cancer targets in three human TNBC cell lines and non-neoplastic MCF10A cells. Red and green bars represent maximum and minimum expression, respectively. High-magnification version is shown in fig. S1. (**B**) Venn diagrams summarize the cancer targets identified by flow cytometric analysis. Each circle represents the number of cancer targets up-regulated in one human TNBC cell line compared to non-neoplastic MCF10A cells. Overlapping sets show the differential expression in two or three comparison pairs, and 16 cancer targets were identified as up-regulated in all three TNBC cell lines compared to MCF10A cells. (**C**) Quantified surface densities of 16 target candidates. Red bars represent the five top candidates that were overexpressed in TNBC cells. (**D**) ICAM1 and EGFR gene expression in human TNBC and MCF10A cells as quantified by qRT-PCR. Significance was measured by one-way analysis of variance (ANOVA) with Bonferroni post hoc test. ***P < 0.001. (**E**) Representative microscopic images of immunofluorescent staining of ICAM1 and EGFR in three human TNBC cell lines and MCF10A cells. Scale bars, 5 μm. DAPI, 4′,6-diamidino-2-phenylindol; FITC, fluorescein isothiocyanate; PE, phycoerythrin. (**F**) FRET analysis of ICAM1 and EGFR colocalization. Significance was measured by one-way ANOVA with Bonferroni post hoc test. FIU, fluorescence intensity unit; NS, not significant; **P < 0.01. (**G**) Correlation between overall survival and ICAM1/EGFR mRNA expression levels in patients with basal-like breast cancer, as shown with Kaplan-Meier analysis (*P < 0.05, log-rank test). Ab, antibody.

Multivalent binding is a proven method to increase selectivity and adhesion strength (*19*–*28*), yet no criteria for the rational selection of targets are described. To address this issue, we designed an unbiased and quantitative method to select and identify optimal target combinations for complementary targeting that could be generally applicable to many cancer types or diseases. Because complementary targeting relies on precisely matched multivalent ligand-receptor interaction, it is crucial to identify two highly overexpressed antigens as target combination and quantify their cell surface density and ratio. Although a number of TNBC targets (e.g., ICAM1, EGFR, CD44, etc.) have been previously identified (*9*, *32*–*35*), their overexpression levels have not been systematically compared and their cell surface ratios are still unknown. We first screened human TNBC cells against a panel of 68 common cancer targets using comparative flow cytometric analyses. In Fig. 1A, fig. S1, and table S1, we quantified the surface protein expression of these cancer targets in three human TNBC cell lines (MDA-MB-231, MDA-MB-436, and MDA-MB-157) in comparison with normal human mammary epithelial MCF10A cells. Of the 68 screened targets, 16 candidates were found to be commonly overexpressed in all three TNBC cell lines and were selected for further evaluation (Fig. 1B). As shown in Fig. 1C, ALCAM (activated leukocyte cell adhesion molecule), ITGA3 (integrin, alpha3), EGFR, ICAM1, and TFRC (transferrin receptor) emerged as the most overexpressed TNBC targets relative to immunoglobulin G (IgG) controls among the 16 candidates. However, ALCAM, ITGA3, and TFRC were also found to be highly expressed in normal MCF10A cells which, if targeted, may cause off-target effects in normal mammary tissues (Fig. 1C). For these reasons, we excluded ALCAM, ITGA3, and TFRC and selected ICAM1 and EGFR as the optimal targets for TNBC complementary targeting due to their high expression in TNBC cells and very low expression in normal cells relative to the other candidates. We recently reported ICAM1 as a novel TNBC target (*8*, *9*); EGFR was also studied as a therapeutic target for TNBC (*32*–*34*). Both ICAM1 and EGFR are molecular targets for FDA-approved drugs (*36*, *37*). However, to date, ICAM1 and EGFR have not been investigated as a target combination for TNBC-specific drug delivery.

We next measured the molecular ratio and organization of ICAM1 and EGFR on TNBC cell surfaces. As shown in table S2, we quantified the surface protein densities of ICAM1 and EGFR on TNBC cells and normal mammary epithelial cells. We also validated the overexpression of ICAM1 and EGFR in TNBC cells at the gene expression level using quantitative real-time polymerase chain reaction (qRT-PCR) (Fig. 1D). Results were consistent with their protein levels on both TNBC and normal cells. We calculated the ICAM1/EGFR surface density ratio for each type of TNBC cell: 4.2:1 for MDA-MB-231, 1.5:1 for MDA-MB-436, and 1.8:1 for MDA-MB-157 (table S2). We selected MDA-MB-231 and MDA-MB-436 for further investigation, as they exhibited the highest and lowest ratio of ICAM1/EGFR. These ICAM1/EGFR surface densities and molecular ratios represent critical design parameters for engineering TNBC-specific DCLs, given that they are the basis for determining the amount and ratio of ICAM1 and EGFR antibodies to be conjugated on the surface of DCLs. This, in turn, facilitates precisely matched, multivalent ligand-receptor interactions with TNBC cells.

Notably, immunofluorescent staining of ICAM1 and EGFR on TNBC cells revealed the overlapped staining of ICAM1 and EGFR (merged fluorescent images in Fig. 1E), indicating that ICAM1 and EGFR are colocalized in close spatial proximity on the cell membrane. The colocalization of two receptors is a key design parameter in engineering of DCLs because complementary targeting requires ICAM1 and EGFR antibodies on the DCL surface to be in contact with both target receptors on the TNBC cell membrane at the same time. Therefore, ICAM1 and EGFR must spatially reside within the distance of the DCL diameter (approximately 130 nm). The colocalization of ICAM1 and EGFR on TNBC cells was also confirmed using a fluorescence resonance energy transfer (FRET) assay. As demonstrated in Fig. 1F, MDA-MB-231, MDA-MB-436, and MCF10A cells were costained with Alexa Fluor 488–ICAM1 antibody (FRET donor: excitation, 495 nm; emission, 515 nm) and Alexa Fluor 555–EGFR antibody (FRET receptor: excitation, 519 nm; emission, 565 nm). FRET signals from the donor-receptor pair were observed on both TNBC cells but were absent in normal MCF10A cells, indicating that ICAM1 and EGFR are present within the Förster radius of 10 nm [the maximum distance for FRET events (*38*)] on TNBC cell membranes.

We also analyzed the potential impact of ICAM1 and EGFR overexpression on the overall survival of patients with basal-like breast cancer (majority are TNBC cases) in a cohort of 25 specimens using the R2: Genomics Analysis and Visualization Platform (https://hgserver1.amc.nl/; Datasheet: Tumor Breast - Bergh - 159 - MAS5.0 - u133a) (*39*). Basal-like breast cancer patients with high expression of both ICAM1 and EGFR demonstrated the worst prognosis (*P* = 0.023, log-rank test; Fig. 1G) relative to overexpression of ICAM1 and EGFR alone. These findings suggest that high expression of ICAM1 in combination with high expression of EGFR may serve as an important clinical biomarker of poor prognosis in patients with basal-like breast cancer.

Although many cell membrane proteins (e.g., ICAM1, EGFR, and CD44) identified from our surface marker screening have previously been reported as promising TNBC targets (*9*, *32*–*35*), the novelty of this surface marker screening study lies in identifying the optimal cancer target combination for dual complementary targeting, which requires a quantitative and systematic comparison of current TNBC antigens. The optimal antigens need to be highly overexpressed and colocalized within the same cell membrane regions of TNBC cells while having minimum expression on normal cells. The quantitative molecular ratio of the dual target combination is also critical for mediating successful dual complementary targeting. Such information has not yet been quantified until this current study.

### Engineering complementary targeting liposomes (DCLs)

Nontargeting liposomal doxorubicin (e.g., Doxil and Myocet) is FDA-approved; these breast cancer nanomedicines exhibit fewer adverse effects and better safety profiles than conventional chemotherapeutics (*31*, *40*, *41*). Unfortunately, these nontargeting liposomes fail to exhibit significantly improved clinical benefits against TNBC due to their limited tumor delivery (*40*–*42*). We reasoned, therefore, that combining our novel complementary targeting strategy with clinically used liposomal doxorubicin would enable a nanotherapeutic to specifically recognize and target TNBC tumors and spare healthy organs and tissues. This approach increases the drug delivery to, and dosage in, tumors, reduces nonspecific uptake, and attenuates adverse effects.

**Fig. 2:**
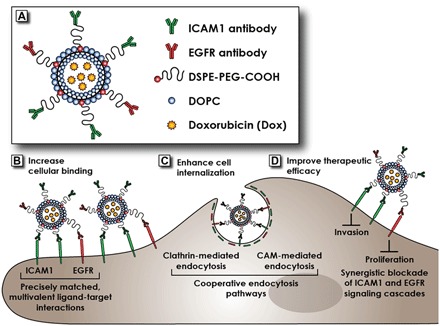
Schematic illustration of DCL structure and biomechanisms of complementary targeting strategy. (**A**) The design of the proof-of-principle binary DCL for TNBC. (**B**) DCL increases cellular binding using precisely matched, multivalent ligand-receptor interactions. (**C**) DCL enhances internalization using cooperative ICAM1 and EGFR endocytosis pathways. (**D**) DCL improves therapeutic efficacy using synergistic blockade of ICAM1 and EGFR signaling cascades.

To test our hypothesis, we designed a proof-of-principle DCL by covalently conjugating both ICAM1- and EGFR-neutralizing antibodies on the surface of liposomal doxorubicin at optimal antibody ratios for different types of TNBC cells (Fig. 2A). For example, 4.2:1 (ICAM1/EGFR antibody) for MDA-MB-231 and 1.5:1 for MDA-MB-436 cells. DCLs were composed of 1,2-dioleoyl-sn-glycero-3-phosphocholine (DOPC) and 1,2-distearoyl-sn-glycero-3-phosphoethanolamine-*N*-[carboxy(polyethylene glycol)-2000] (DSPE-PEG-COOH) (95/5, mol/mol). We characterized the size and monodispersity of synthesized DCLs by dynamic light scattering measurements (table S3 and fig. S2). All DCLs and control liposomes exhibited uniform hydrodynamic radii of approximately 130 ± 30 nm and z potentials between −6 and −10 mV. The ICAM1/EGFR antibody ratios conjugated on DCL surfaces were also measured and are close to their theoretical values (table S4).

### Complementary targeting enhances liposome binding to TNBC cells

**Fig. 3:**
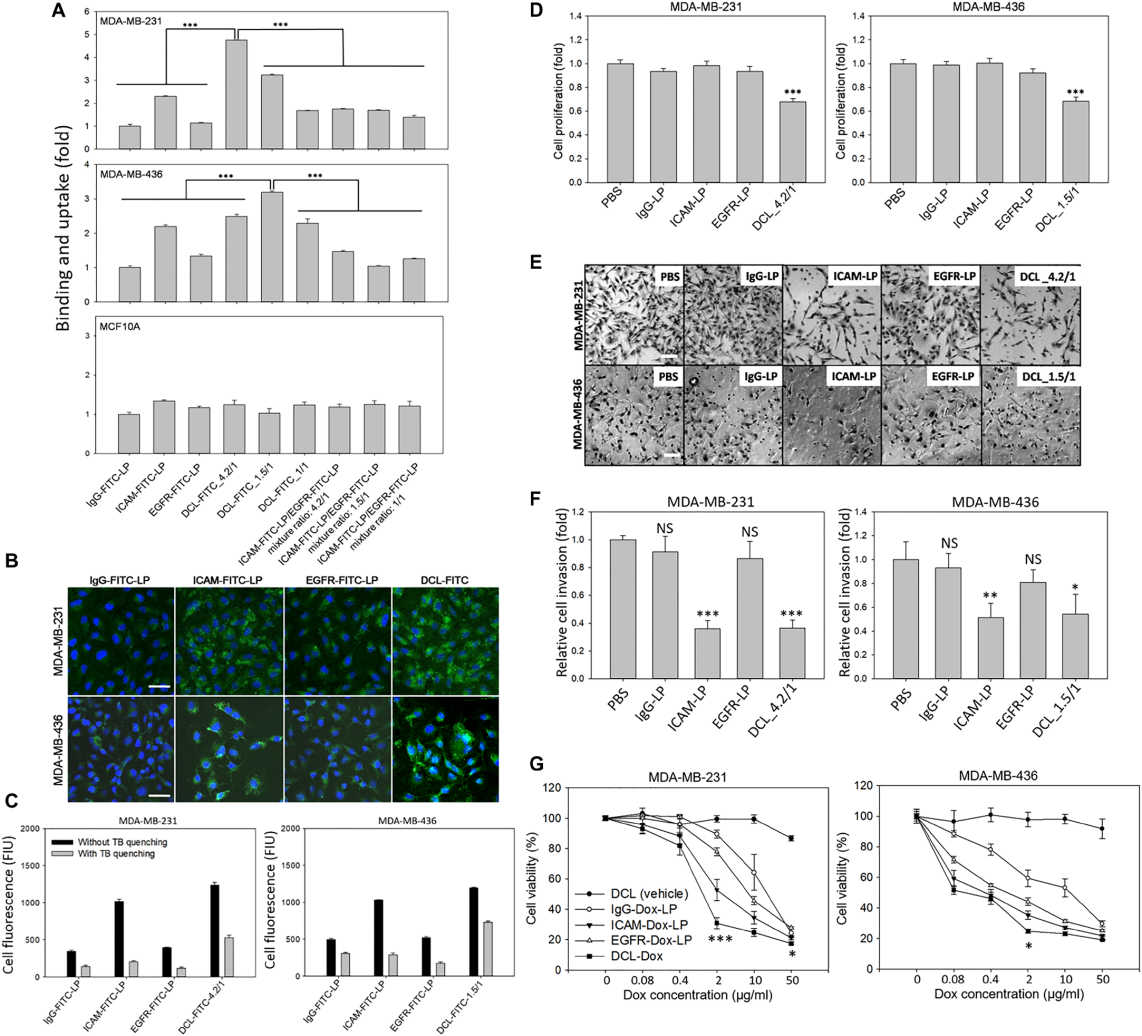
DCL increases in vitro TNBC targeting and antitumor activities. (**A**) In vitro cellular binding and uptake of DCL-FITC and controls in human TNBC and MCF10A cells were determined by flow cytometry in reference to IgG-FITC-LP. (**B**) Representative fluorescent images showing TNBC-specific cellular binding and uptake of DCL-FITCs in TNBC cells in comparison with IgG-FITC-LP, ICAM-FITC-LP, and EGFR-FITC-LP. Scale bars, 20 μm. (**C**) Internalization ratios of DCL-FITC and controls were determined by a trypan blue (TB) quenching assay. Black bars (without trypan blue quenching) represent total cellular fluorescence from both extracellular and internalized liposomes, and gray bars (with trypan blue quenching) represent cellular fluorescence from internalized liposomes only. (**D**) Quantified analysis of therapeutic efficacies of DCL (vehicle without Dox) and controls on TNBC cell proliferation. Representative microscope images (**E**) and quantitative analysis (**F**) of invaded TNBC cells in a transwell invasion assay. Scale bars, 50 μm. (**G**) In vitro cytotoxicity of DCL-Dox was evaluated for MDA-MB-231 and MDA-MB-436 cells by Dojindo cell viability assay in reference to DCL (vehicle without Dox). Significance was measured by one-way ANOVA with Bonferroni post hoc test. *P < 0.05; **P < 0.01; ***P < 0.001.

TNBC binding and uptake of DCLs were determined by both flow cytometry and immunofluorescent staining. As demonstrated in Fig. 3A, we treated MDA-MB-231 and MDA-MB-436 cells with fluorescein isothiocyanate (FITC)–labeled DCLs with different ICAM1/EGFR antibody ratios (DCL-FITC_4.2/1, _1.5/1, and _1/1), FITC-labeled ICAM1 or EGFR single-targeting liposomes (ICAM-FITC-LP or EGFR-FITC-LP), or nontargeting IgG-FITC-LP in the presence of serum [10% fetal bovine serum (FBS)]. DCL-FITC_4.2:1 (ICAM1/EGFR antibody ratio 4.2/1, optimized for MDA-MB-231cells) exhibited a 4.7-fold increase in binding with MDA-MB-231 cells as compared to IgG-FITC-LP, significantly higher than other tested DCLs and ICAM1 or EGFR single-targeting liposomes. Consistently, DCL-FITC_1.5/1 (ICAM1/EGFR antibody ratio 1.5/1, optimized for MDA-MB-436 cells) exhibited the highest cellular binding with MDA-MB-436 cells (Fig. 3A). Meanwhile, we observed no obvious changes in cellular binding in normal MCF10A cells treated with DCLs or control liposomes due to their lack of either ICAM1 or EGFR expression. We also observed increased cellular binding with DCL-FITC with immunofluorescent staining (Fig. 3B). These results demonstrated that the ICAM1/EGFR antibody ratio plays a critical role in regulating multivalent ligand-receptor interactions between DCLs and TNBC cells. As illustrated in Fig. 2B, only when the ICAM1/EGFR antibody ratio on DCLs precisely complements the ICAM1/EGFR expression ratio on TNBC cells does the multivalent ligand-receptor interaction reach its maximum efficiency and generate the strongest cooperative adhesion specifically toward TNBC cells, thereby significantly promoting TNBC cellular binding.

We also treated cells with single-targeting liposomes mixed at different ratios. It is important to note that simply mixing ICAM1 and EGFR single-targeting liposomes at matching molar ratios (e.g., 4.2/1, 1.5/1, and 1/1) did not improve their cellular binding in comparison with DCLs (Fig. 3A). This is due to the fact that the mixture of single-targeting liposomes alone lacks the multivalent ligand-receptor interaction toward TNBC cells and also causes steric hindrance as both ICAM-FITC-LP and EGFR-FITC-LP compete to bind colocalized ICAM1 and EGFR in the same cell surface regions.

### Complementary targeting significantly enhances liposome internalization in TNBC cells

The advantages of complementary targeting are not limited to the increased TNBC cellular binding. We also observed that this strategy substantially enhanced TNBC cell internalization of liposomes via cooperative endocytosis pathways (Fig. 2C). It is known that EGFR internalization mainly depends on clathrin-mediated endocytosis (*43*), while ICAM1 internalization relies on an alternative cell adhesion molecule (CAM)–mediated pathway (*44*). We reasoned that DCLs may simultaneously bind and activate both ICAM1 and EGFR internalization pathways and enter TNBC cells via a synergy of clathrin- and CAM-mediated endocytosis. To test this hypothesis, we performed trypan blue quenching assays on DCL-FITC–treated TNBC cells to block the extracellular fluorescence from bound and non-internalized DCL-FITCs and calculated the internalization ratio of DCL-FITCs by dividing the cellular fluorescence of internalized DCL-FITCs by the total cellular fluorescence composed of both extracellular and internalized DCL-FITCs (Fig. 3C). Unexpectedly, ICAM1- or EGFR single-targeting liposomes, which exhibited increased cellular binding (Fig. 3, A and B), bound to TNBC cell surfaces via ICAM1 or EGFR antibody-antigen interactions and were not effectively internalized by TNBC cells. This may be due to the limited efficacy of the ICAM1 or EGFR single endocytosis pathway. In contrast, DCL-FITCs significantly restored the internalization ratio back to 42.7% for MDA-MB-231 cells and 60.9% for MDA-MB-436 cells while maintaining their highly specific TNBC cellular binding (Fig. 3C). The IgG group demonstrated a high internalization ratio (40 to 60%) due to its low affinity for the cell surface compared to other groups. These results demonstrated that the complementary targeting strategy enables liposomes to enter TNBC cells more efficiently via cooperative endocytosis pathways. Naturally occurring proteins [e.g., LRP1 (low density lipoprotein receptor-related protein 1)] are known to harness these cooperative endocytosis pathways to enhance their cell entry (*45*, *46*).

We hypothesize that synthetic nanocarriers can exploit multiple endocytosis pathways to improve cell internalization. The detailed biomechanism(s) of this synergy between clathrin and CAM-mediated endocytosis pathways merits further investigation.

### Complementary targeting cooperatively blocks ICAM1 and EGFR signaling cascades

We engineered our DCLs with ICAM1- and EGFR-neutralizing antibodies that could simultaneously block ICAM1 and EGFR signaling cascades in TNBC cells (Fig. 2D). The EGFR-neutralizing antibody cetuximab is an FDA-approved antitumor agent for treating a variety of metastatic tumors (*47*). ICAM1-neutralizing antibodies, enlimomab and BI-505, have shown promising antitumor activities against many cancers (*30*, *48*, *49*). We therefore reasoned that our DCL is not only a drug delivery nanocarrier but also a TNBC-targeted therapeutic agent that synergistically inhibits both ICAM1 and EGFR pathways in TNBC cells and therefore blocks multiple processes during cancer progression. We therefore investigated the impact of the DCL vehicle (without Dox) on both TNBC cell proliferation and invasion. DCLs exhibited a 30 to 40% inhibitory effect on TNBC cell proliferation in vitro in both MDA-MB-231 and MDA-MB-436 cells (Fig. 3D). Moreover, as presented in Fig. 3 (E and F), DCLs exhibited potent inhibitory activity against TNBC cell invasion. The number of invaded MDA-MB-231 and MDA-MB-436 cells was significantly reduced by 64 and 46%, respectively, by DCL treatment in comparison with phosphate-buffered saline (PBS) controls. Notably, we observed a similar inhibitory effect with ICAM-LP but not with EGFR-LP, indicating that the inhibitory function of DCLs against cell invasion may be attributed to the blockade of the ICAM1 pathway rather than the EGFR pathway. This inhibitory effect was consistent with previous studies using free ICAM1-neutralizing antibodies (*49*). On the basis of these data, we postulated that ICAM1-neutralizing antibodies of DCLs may work as bioactive therapeutic agents against TNBC progression and metastasis via blocking its pathway.

The potent inhibitory effects of this DCL vehicle on TNBC cell proliferation and invasion may further synergize with its chemotherapeutic payloads (e.g., doxorubicin) to generate maximal therapeutic benefits in vivo against TNBC progression and metastasis. To test this hypothesis, we loaded DCLs with doxorubicin (DCL-Dox), a commonly used breast cancer chemotherapy drug, and evaluated its half maximal inhibitory concentration (IC_50_) in two human TNBC cell lines. Doxorubicin-loaded DCL_4.2/1 (DCL-Dox_4.2/1) (optimized for MDA-MB-231 cells) showed significantly improved cytotoxicity against MDA-MB-231 cells, 13-fold higher than the cytotoxicity from IgG-conjugated PEGylated liposomal doxorubicin (IgG-Dox-LP) (Fig. 3G). The quantified IC_50_ for IgG-Dox-LP, ICAM-Dox-LP, EGFR-Dox-LP, and DCL-Dox_4.2/1 in MDA-MB-231 cells were 11.7, 2.4, 4.8, and 0.9 μg/ml, respectively. We also observed a 1MDA-MB-436 cells, achieving the lowest IC_50_ of DCL-Dox_1.5/1 (0.04 μg/ml) compared with IgG-Dox-LP (3.74 μg/ml), ICAM-Dox-LP (0.08 μg/ml), and EGFR-Dox-LP (0.23 μg/ml). In summary, DCL-Dox exhibited the lowest IC_50_ in both MDA-MB-231 and MDA-MB-436 cells due to their complementary targeting capability.

### DCL inhibits orthotopic TNBC tumor growth and metastasis

**Fig. 4:**
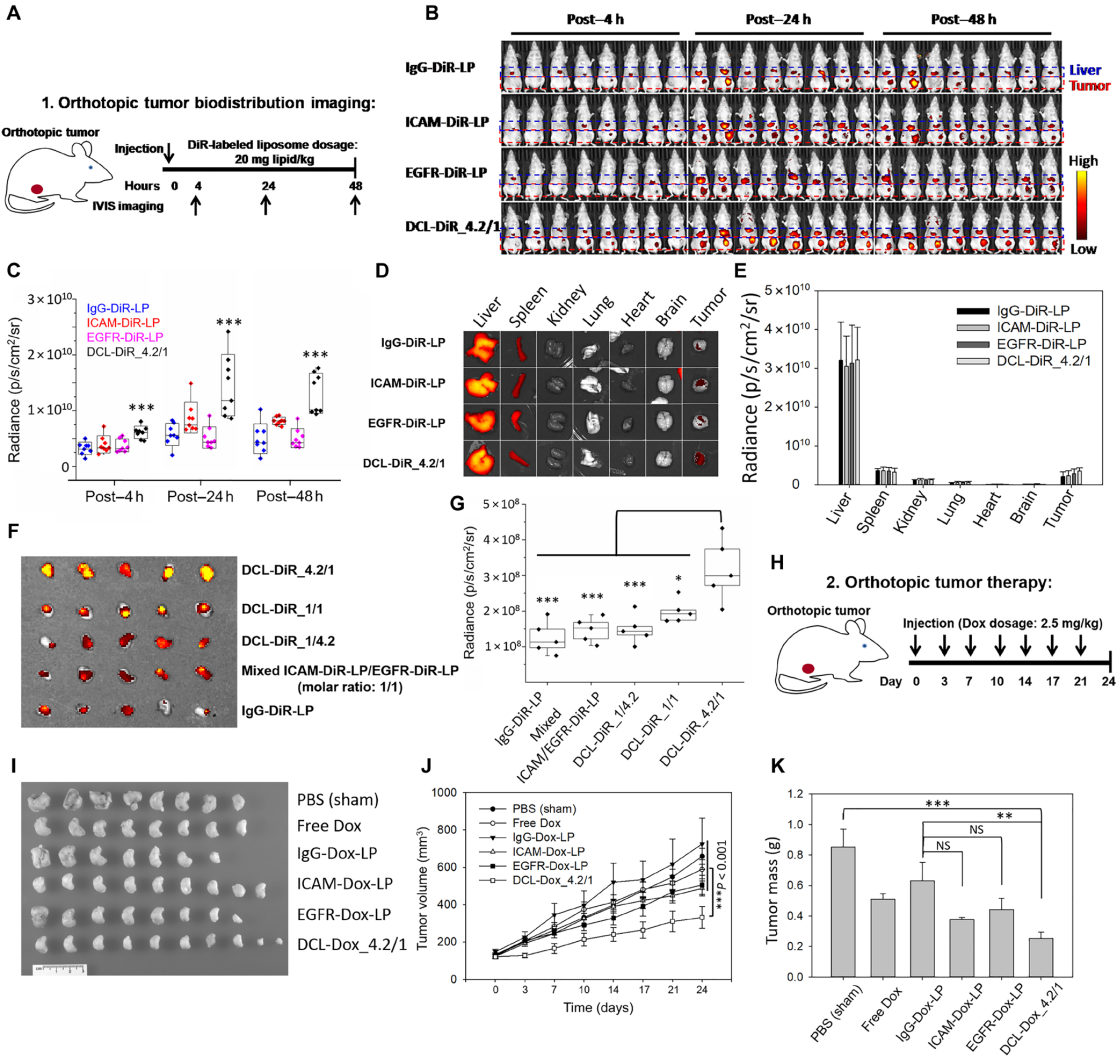
DCL-Dox inhibits orthotopic tumor growth and metastasis. (**A**) Schematic design of orthotopic tumor biodistribution imaging. (**B**) In vivo NIR fluorescent images of nude mice at 4, 24, and 48 hours after the administration of IgG-DiR-LP, ICAM-DiR-LP, EGFR-DiR-LP, and DCL-DiR_4.2/1. *n* = 8 per group. (**C**) Quantitative analysis of in vivo tumor accumulation of DCL-DiR_4.2/1 and control liposomes. (**D**) Representative ex vivo NIR fluorescent images of organs (liver, spleen, kidney, lung, heart, and brain) and excised tumors. (**E**) Biodistribution of administrated agents in different organs and tumors after 48 hours. (**F**) TNBC tumor specificity of DCLs with three distinct ratios (4.2/1, 1/1, and 1/4.2, mol/mol) and a mixture of two single-targeting liposomes (ICAM-DiR-LP and EGFR-DiR-LP, 1/1, mol/mol) in the orthotopic TNBC model (MDA-MB-231) (*n* = 5 per group). (**G**) Tumor accumulation of liposomes was determined at 48 hours after injection by NIR fluorescent intensity (*n* = 5 per group). (**H**) Schematic design of orthotopic tumor therapy model. (**I**) Image of excised orthotopic TNBC tumors from mice treated with PBS (sham), free Dox, IgG-Dox-LP, ICAM-Dox-LP, EGFR-Dox-LP, or DCL-Dox_4.2/1 under a 21-day treatment regimen (*n* = 7 to 10 per group). (**J**) Tumor progression was closely monitored by weekly tumor volume measurement using caliper. (**K**) Tumor mass at end point (day 24) was quantified in weight. Significance was measured by one-way ANOVA (C, E, G, and K) or two-way ANOVA (J) with Bonferroni post hoc test. *P < 0.05; **P < 0.01; ***P < 0.001.

We first evaluated the in vivo tumor-targeting activity of DCLs using near-infrared (NIR) fluorescent imaging in an orthotopic TNBC tumor model (Fig. 4A). We labeled DCL_4.2/1 with 1,1′-dioctadecyl-3,3,3′,3′-tetramethylindotricarbocyanine iodide (DiR), a NIR lipid dye, (DCL-DiR_4.2/1) and intravenously injected it into luciferase-labeled MDA-MB-231 (MDA-MB-231-Luc) tumor–bearing mice. We used IgG-DiR-LP, ICAM-DiR-LP, and EGFR-DiR-LP as controls. We performed in vivo NIR imaging at 6, 24, and 48 hours after injection. Among four tested formulations, the DCL-DiR_4.2/1 group demonstrated the highest tumor accumulation at all time points (Fig. 4B). Quantified NIR signals confirmed that the tumor accumulation of DCL-DiR_4.2/1 was 2.8-fold higher than that of IgG-DiR-LP at 24 hours after a single tail vein administration. We quantified the tumor accumulation percentage of DCL-DiR_4.2/1 by dividing the tumor NIR signal (the NIR signal integrated over the tumor area) by the total NIR signal of administered DiR-labeled liposomes. We quantified the average percentage of DCL-DiR_4.2/1 accumulated in the tumors as 3.3% in comparison with 1.2% of IgG-DiR-LP, 2.0% of ICAM-DiR-LP, and 1.3% EGFR-DiR-LP (Fig. 4C). We evaluated the biodistribution of DCL-DiR_4.2/1 using ex vivo quantification of NIR signals in six organs and tumors excised from mice at 48 hours (Fig. 4, D and E). Correlating with in vivo whole mouse imaging data, DCL-DiR_4.2/1 accumulated in excised tumors approximately 1.7-fold higher than that of IgG-DiR-LP (Fig. 4E). These results demonstrated that complementary targeting is more effective than conventional single-targeting approaches in recognizing and targeting TNBC tumors in vivo.

The dual-targeting strategy is known to enhance tumor uptake of nanomedicines (*22*–*28*); however, the impact(s) of optimizing the targeting ligand ratio on in vivo delivery efficacy is still unclear, and, to date, the target ligand ratio is not used as a design parameter for dual-targeted nanomedicines. Thus, we systematically compared the in vivo tumor uptake of DCLs with three distinct ICAM1/EGFR antibody ratios (4.2/1, 1/1, 1/4.2, mol/mol) in an orthotopic TNBC model (MDA-MB-231-Luc) as described above. A mixture of two single-targeting liposomes (ICAM-DiR-LP and EGFR-DiR-LP, 1/1, mol/mol) and nonspecific IgG-DiR-LP were used as controls. As shown in Fig. 4 (F and G), DCL-DiR_4.2/1, with the precise ICAM1/EGFR antibody ratio to complement the antigen overexpression in MDA-MB-231-Luc tumors, exhibited over 2.5-fold increased tumor uptake than IgG-DiR-LP, significantly higher than other DCLs with equivalent and reversed antibody ratios (1.6-fold for DCL-DiR_1/1 and 1.2-fold for DCL-DiR_1/4.2). This in vivo tumor uptake of DCL-DiR_4.2/1 was also found being consistent with previous biodistribution studies (Fig. 4C). The mixture of ICAM-DiR-LP and EGFR-DiR-LP did not effectively improve the in vivo tumor uptake due to their lack of multivalent ligand-receptor interactions. To our knowledge, the in vivo data presented here are the first experimental evidence of targeting ligand ratio regulating in vivo tumor uptake for “dual-ligand targeting” nanomedicines.

We next examined the therapeutic efficacy of DCL-Dox_4.2/1 in inhibiting orthotopic TNBC tumor growth and metastasis (Fig. 4H). Doxil (PEGylated liposomal doxorubicin) is an FDA-approved nanomedicine but failed to demonstrate enough therapeutic efficacy against TNBC (*31*, *40*). We hypothesized that combining our complementary targeting with clinically used Doxil could increase drug delivery to TNBC tumors and improve therapeutic efficacy. To evaluate the effectiveness of this complementary targeting strategy, we chose to use nontargeting IgG-Dox-LP as a surrogate of Doxil to compare with DCL-Dox_4.2/1 because these formulations share the same physiochemical properties except their targeting activity. MDA-MB-231-Luc tumor–bearing mice were randomly divided into six groups and received treatment of PBS (sham), free doxorubicin (free Dox), IgG-Dox-LP, ICAM-Dox-LP, EGFR-Dox-LP, or DCL-Dox_4.2/1, respectively, at a Dox dosage of 2.5 mg/kg via retro-orbital injection. As shown in Fig. 4 (I and J), after a 21-day treatment regimen, DCL-Dox_4.2/1 exhibited the highest inhibitory effect on TNBC tumor growth among all tested groups. The quantified tumor mass revealed that DCL-Dox significantly reduced TNBC tumor growth by 70.3%, approximately threefold more efficient than IgG-Dox-LP (Fig. 4K). Furthermore, as shown in Table 1, DCL-Dox_4.2/1 substantially inhibited spontaneous metastasis compared to other groups (1 of 10 mice versus 5 of 9 to 8 of 8 mice). Overall, DCL-Dox_4.2/1 exhibited significantly higher biodistribution in the tumor, lower tumor growth, and inhibited metastasis relative to all controls.

**Table 1:** Summary of metastasis formation in TNBC orthotopic and lung metastasis models.

**Metastatic site**	**PBS (sham)**	**Free Dox**	**IgG-Dox-LP**	**ICAM-Dox-LP**	**EGFR-Dox-LP**	**DCL-Dox_4.2/1**
**Orthotopic breast tumor model**						
Brain	0/8	0/8	0/7	0/9	0/8	0/10
Lung	1/8	0/8	0/7	0/9	1/8	0/10
Heart	0/8	0/8	0/7	0/9	0/8	0/10
Liver	3/8	0/8	2/7	0/9	1/8	0/10
Spleen	3/8	0/8	2/7	1/9	1/8	1/10
Kidney	0/8	0/8	0/7	0/9	0/8	0/10
Right hind limb (tumor bearing)	6/8	8/8	5/7	4/9	5/8	1/10
Left hind (normal)	1/8	0/8	0/7	2/9	1/8	0/10
**Total**	7/8	8/8	6/7	5/9	5/8	1/10
**Lung metastasis model**						
Brain	0/8	0/8	0/8	0/8	0/8	0/8
Lung	8/8	5/8	6/8	2/8	6/8	0/8
Heart	0/8	0/8	0/8	0/8	0/8	0/8
Liver	2/8	0/8	1/8	0/8	0/8	0/8
Spleen	1/8	0/8	0/8	0/8	0/8	0/8
Kidney	0/8	0/8	0/8	0/8	0/8	0/8
Hind limbs (normal)	0/8	0/8	0/8	0/8	0/8	0/8
**Total**	8/8	5/8	6/8	2/8	6/8	0/8

### DCL inhibits TNBC lung metastasis

**Fig. 5:**
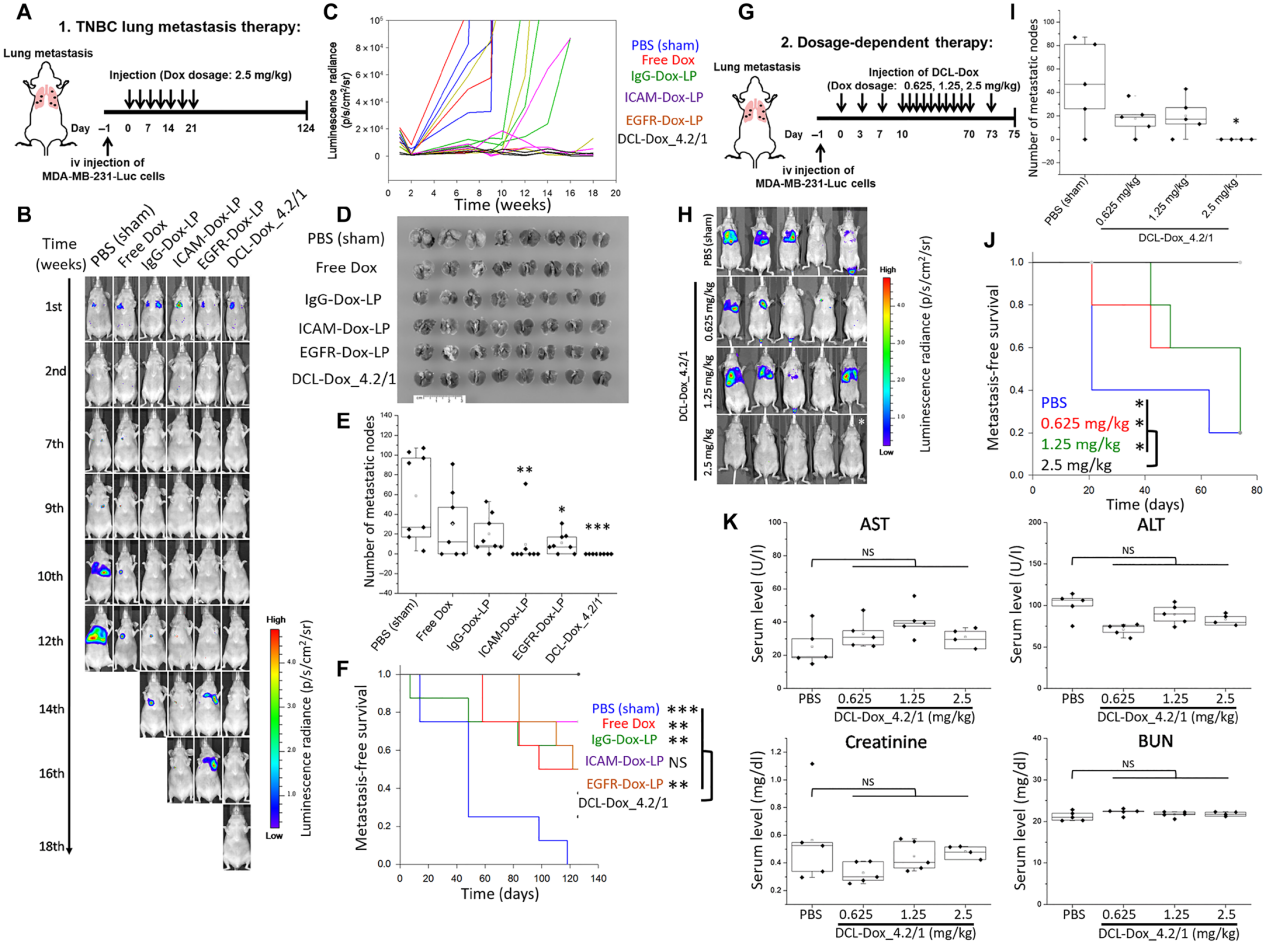
DCL-Dox inhibits TNBC lung metastasis and improves survival. (**A**) Schematic design of TNBC lung metastasis therapy. (**B**) Representative bioluminescence images of lung metastasis at different time points in mice treated with the following agents: PBS (sham), free Dox, IgG-Dox-LP, ICAM-Dox-LP, EGFR-Dox-LP, or DCL-Dox_4.2/1 (*n* = 8 per group). (**C**) Representative tumor progression curves as depicted from in vivo bioluminescence signal intensity (*n* = 3 per group). (**D**) Size and morphology of lungs excised from mice in different treatment groups. (**E**) Quantification of metastasis node numbers on excised lungs from mice in different treatment groups. (**F**) Metastasis-free survival of mice in DCL-Dox and control groups as displayed by Kaplan-Meier curves (log-rank test). (**G**) Schematic design for dosage-dependent therapy. iv, intravenous. (**H**) In vivo bioluminescence images of mice in the dosage-dependent study. Tumor-bearing mice were treated with DCL-Dox_4.2/1 at different dosages and imaged at day 74 or an earlier sacrifice date (*n* = 5 per group). “*” indicates the mouse sacrificed at day 22 due to blindness caused by retro-orbital injection). (**I**) Quantification of metastasis node numbers on excised lungs in the dosage-dependent study. (**J**) Metastasis-free survival of mice in the dosage-dependent study as displayed by Kaplan-Meier curves (log-rank test). (**K**) Serum levels of aspartate aminotransferase (AST), alanine aminotransferase (ALT), creatinine, and blood urea nitrogen (BUN) (*n* = 4 to 5 per group). Significance was measured by one-way ANOVA with Bonferroni post hoc test (E, I, and K). *P < 0.05; **P < 0.01; ***P < 0.001.

To extend the application of complementary targeting strategy to metastatic TNBC, we examined the antitumor activity of DCL-Dox in a lung metastasis model, which is known to be more aggressive and more refractory to conventional chemotherapy than an orthotopic tumor model (*50*–*53*). We generated TNBC lung metastases by tail vein administration of MDA-MB-231-Luc cells (Fig. 5A). After confirming the formation of lung metastasis by in vivo bioluminescence imaging (Fig. 5B), mice were randomly divided into the same treatment groups used in the orthotopic model and administered via retro-orbital injection. After a 21-day treatment regimen, lung metastasis in each group was closely monitored by weekly bioluminescence imaging up to 124 days (Fig. 5, B and C). As shown in Fig. 5 (B and C), DCL-Dox completely inhibited the progression of TNBC lung metastasis compared to the other groups. None of the mice treated with DCL-Dox_4.2/1 developed lung metastases, whereas six of eight mice in the nontargeting IgG-Dox-LP and EGFR-Dox-LP group developed metastases (Table 1). ICAM-Dox-LPs also exhibited a slightly lower inhibitory activity (two of eight mice) than DCL-Dox, which correlates with the in vitro cell invasion studies (Fig. 3H). The DCL-Dox_4.2/1 complete inhibition of TNBC metastasis formation on excised lungs was confirmed in Fig. 5 (D and E). We further found that this potent metastasis inhibitory activity of DCL-Dox_4.2/1 led to significant survival benefits. As shown in Fig. 5F, DCL-Dox substantially improved metastasis-free survival in comparison with all groups except ICAM-Dox-LP.

### Determination of the optimal dosage for DCL therapy

We further performed a dosage-dependent study to determine the minimum effective dosage of DCL-Dox treatment (Fig. 5G). Mice with MDA-MB-231 lung metastases were treated with PBS (sham) or DCL-Dox_4.2/1 at three dosages (0.625, 1.25, and 2.5 Dox mg/kg) for up to 75 days. DCL-Dox at the dosages of 0.625 and 1.25 mg/kg did not inhibit lung metastasis as effectively as DCL-Dox at the dosage of 2.5 mg/kg (Fig. 5, H and I). Kaplan-Meier survival analysis further confirmed the significantly increased survival benefit of the 2.5 mg/kg dosage compared to the lower dosages (Fig. 5J). Thus, DCL-Dox_4.2/1 at the dosage of 2.5 mg/kg Dox was considered to be the optimal dosage for treating metastatic MDA-MB-231 tumors.

We evaluated the chronic liver and renal toxicity of DCL-Dox_4.2/1 treatment via blood chemistry analysis. At the end of the DCL-Dox_4.2/1 dosage-dependent study (day 75), we collected the serum from each dosage group and measured aspartate aminotransferase (AST) and alanine aminotransferase (ALT) levels to evaluate liver toxicity. As shown in Fig. 5K, among all DCL-Dox_4.2/1 dosages, none of them, including the highest one, induced any elevation in either AST or ALT levels compared with the PBS group. Similarly, we evaluated the renal toxicity of DCL-Dox_4.2/1 by measuring creatinine and blood urea nitrogen (BUN) levels, and we observed no renal toxicity among these DCL-Dox_4.2/1 dosage groups (Fig. 5K). The highest Dox dosage at 2.5 mg/kg for 75 days in mice is equivalent to a Dox cumulative dosage of 1760 mg/m^2^ in human, which is close to the Dox life time cumulative dosage of 2220 mg/m^2^ in human (*54*). These in vivo data demonstrate that DCL-Dox_4.2/1 at 2.5 mg/kg dosage exhibited the highest inhibitory activity against primary and metastatic TNBC tumors while exhibiting no systemic toxicity.

In summary, we have demonstrated that complementary targeting is a highly precise and effective strategy to recognize and target TNBC tumors both in vitro and in vivo. We have engineered a dual complementary targeting, Dox-encapsulating liposome that significantly inhibits TNBC tumor progression and metastasis in both orthotopic tumor and lung metastasis models. In addition, we have provided an unbiased and quantitative screening method to identify optimal candidates for dual-targeted drug delivery, which provides the opportunity for other investigators to readily apply this complementary targeting strategy to the design of nanomedicines to treat other cancers or diseases. We have elucidated the biomechanisms by which complementary targeted nanotherapeutics interact with biological systems, providing tunable parameters to optimize tumor specificity and therapeutic efficacy for multivalent nanomedicines.

## MATERIALS AND METHODS

### Materials

Dulbecco’s PBS, 4′,6-diamidino-2-phenylindole (DAPI), 0.25% trypsin/2.6 mM EDTA solution, Gibco Dulbecco’s modified Eagle’s medium (DMEM), Gibco DMEM/F12 (1:1), and Gibco 0.4% trypan blue solution were purchased from Invitrogen (Carlsbad, CA, USA). 1-Ethyl-3-(3-dimethylaminopropyl) carbodiimide hydrochloride (EDC), *N*-hydroxysuccinimide (NHS), bovine serum albumin (BSA), anhydrous dimethyl sulfoxide (DMSO), Dox, FITC-dextran [molecular weight (MW), 10 kDa], AST activity assay kit, ALT activity assay kit, creatinine activity assay kit, and urea activity assay kit were purchased from Sigma-Aldrich (St. Louis, MO, USA). Corning BioCoat Matrigel Invasion Chamber with BD Matrigel Matrix, Lab-Tek II Chamber Slide System, formaldehyde, chloroform, anhydrous ethanol (EtOH), Slide-A-Lyzer dialysis cassette [molecular weight cut-off (MWCO), 10 kDa], DiR, and Diff-Quik Stain Set were purchased from Thermo Fisher Scientific (Pittsburgh, PA, USA). Mouse antihuman ICAM1-neutralizing antibody (clone BBIG-I1) and IgG isotype were purchased from R&D Systems (Minneapolis, MN, USA). Phycoerythrin (PE)–conjugated mouse/rat antihuman antibodies against 68 cancer target candidates (table S1), FITC-ICAM1 antibody, Alexa Fluor 488–ICAM1 antibody, and FITC- and PE-conjugated mouse IgG isotypes were purchased from BioLegend (San Diego, CA, USA). Mouse antihuman EGFR-neutralizing antibody (clone LA1) and Alexa Fluor 555–EGFR antibody were purchased from EMD Millipore (Billerica, MA, USA). DOPC and DSPE-PEG-COOH were purchased from Avanti Polar Lipids (Alabaster, AL, USA). Quantum Simply Cellular microbeads were purchased from Bangs Laboratory (Fishers, IN, USA). Qiagen RNeasy Mini Kit was purchased from Qiagen (Germantown, MD, USA). FLOAT-A-LYZER G2 dialysis tubing (MWCO, 1000 kDa) was purchased from Spectrum Laboratories (Rancho Dominguez, CA, USA). Two-micrometer borosilicate beads were purchased from Thomas Scientific (Swedesboro, NJ, USA). Dojindo cell counting kit was purchased from Dojindo Molecular Technologies (Rockville, MD, USA). BD Vacutainer was purchased from Becton Dickinson (Franklin Lakes, NJ, USA).

### Cell culture

Three human TNBC cell lines (MDA-MB-231, MDA-MB-436, and MDA-MB-157) and one human non-neoplastic mammary epithelial cell line (MCF10A) were used in the presented study. All four cell lines were purchased from American Type Culture Collection (Manassas, VA, USA). MDA-MB-231, MDA-MB-436, and MDA-MB-157 cells were cultured in DMEM, and MCF10A cells were cultured in DMEM/F12 (1:1), with all recommended supplements. All cells were maintained at 37°C in a humidified incubator with 5% CO_2_. Luciferase-labeled MDA-MB-231 (MDA-MB-231-Luc-D3H2LN) cells were purchased from PerkinElmer (Hopkinton, MA, USA) and cultured using the same condition as MDA-MB-231 cells.

### Screening and identification of optimal targets for complementary targeting

Cell membrane expression of molecular target candidates was evaluated using a BD FACSCalibur Flow Cytometer (BD Biosciences, San Jose, CA, USA), as described previously (*7*). Briefly, 10^6^ cells were collected and rinsed twice through suspension-spin cycles. Cells were blocked by 1% BSA in PBS for 30 min in an ice bath. After BSA blocking, cells were incubated with PE-conjugated antibodies for 1 hour at room temperature (RT). Cells were rinsed twice with 1% BSA in PBS, resuspended in PBS, and evaluated by flow cytometry. Density of molecular targets on the cell surface was determined with reference to Quantum Simply Cellular microbeads, using the protocol provided by the manufacturer.

### Quantification of gene expression

Gene expression levels of ICAM1 and EGFR in TNBC cells were characterized using qRT-PCR. Cells were cultured overnight at 3 × 10^5^ cells per well in a six-well cell culture plate. Cells were then removed from each well by incubating with a 0.25% trypsin/2.6 mM EDTA solution for 3 min. The cells were washed three times with PBS. RNA was extracted, purified using the Qiagen RNeasy Mini Kit, and quantified using a SpectraMaxPlus 384 UV-Visible Spectrophotometer (Molecular Devices Corporation, Sunnyvale, CA, USA). Reverse transcription was conducted using the Applied Biosystems TaqMan RT protocol. Detection and quantification of mRNA were performed using the StepOnePlus Real-Time PCR System (Applied Biosystems, Carlsbad, CA, USA). All PCR samples were referenced to the gene expression level of glyceraldehyde 3-phosphate dehydrogenase.

### Immunofluorescent staining

Twenty thousand cells were seeded in a Lab-Tek II Chamber Slide System with 2-ml medium overnight at 37°C. After medium was removed, cells were rinsed twice with PBS and fixed with 4% formaldehyde in PBS at RT for 10 min, followed by washing with PBS. Samples were blocked with 1% BSA in PBS for 30 min in an ice bath. After BSA blocking, samples were costained with FITC-conjugated ICAM1 antibody and PE-conjugated EGFR antibody for 1 hour and rinsed with PBS. DAPI was used to stain the cell nucleus. Immunofluorescent stained samples were dried overnight in the dark and used for fluorescent microscope imaging. Samples were examined under a Leica TCS SP5 confocal fluorescent microscope (Leica Microsystems, Buffalo Grove, IL, USA).

### FRET assay

The FRET assay was performed on live MDA-MB-231, MDA-MB-436, and MCF10A cells. Cells (10^4^) were seeded in each well of a 96-well plate and grown overnight. Cells were washed twice with PBS and incubated with PBS, Alexa Fluor 488–ICAM1 antibody (donor), Alexa Fluor 555–EGFR antibody (receptor), or a mixture of Alexa Fluor 488–ICAM1 antibody and Alexa Fluor 555–EGFR antibody (donor + receptor, 1:1) at a final antibody concentration of 1 μg/10^6^ cells for 45 min at 37°C. After staining, cells were washed twice with PBS, and their FRET signals were measured at the donor’s excitation wavelength of 495 nm and the receptor’s emission wavelength of 565 nm using a SpectraMaxPlus 384 UV-Visible Spectrophotometer (Molecular Devices Corporation, Sunnyvale, CA, USA).

### Preparation of doxorubicin-encapsulating DCL (DCL-Dox)

DCL-Dox was prepared by the extrusion method as described previously with modifications (*7*, *8*, *10*, *11*). Briefly, a lipid formulation consisted of DOPC:DSPE-PEG-COOH (95:5, mol/mol) was used to prepare liposomes. Lipid mixture (50 μmol) was solubilized in chloroform and dried under a dry nitrogen stream. The resulting lipid film was dissolved in 1 ml of DMSO:EtOH (7:3, v/v). The lipid solution was injected into 9 ml of 240 mM sodium sulfate in PBS (pH 7.4) while being rigorously agitated to yield a 5 mM lipid solution. After 10 freeze-thaw cycles, lipid solution was extruded via a Northern Lipids Extruder with a 100-nm polycarbonate nanoporous membrane. After extrusion, the liposome solution was dialyzed in PBS (pH 7.4) using a Slide-A-Lyzer dialysis cassette (MWCO, 20 kDa) overnight at RT. Then, Dox was added to liposome solution to reach a final concentration of 1 mg/ml and incubated for 6 hours to facilitate active loading. The resulting Dox-encapsulating liposome solution was dialyzed in PBS (pH 7.4) using a Slide-A-Lyzer dialysis cassette (MWCO, 20 kDa) overnight at RT.

The surface of DCL-Dox was modified with ICAM1- and EGFR-neutralizing antibodies at optimal ratios via the DSPE-PEG-COOH anchor. EDC (2 mg) and NHS (3 mg) were mixed with 1 mmol of lipid (liposomes) in PBS (pH 7.4) and incubated for 6 hours at RT. A Slide-A-Lyzer dialysis cassette (MWCO, 20 kDa) was used to remove unreacted EDC and NHS. Next, ICAM1- and EGFR-neutralizing antibodies at different molecular ratios (1/0, 0/1, 4.2/1, 1.5/1, and 1/1) or the IgG isotype were added to EDC-modified liposomes at a molar ratio of 1:1000 (antibody/phospholipid) and incubated overnight at RT. Unreacted antibodies were removed by using a FLOAT-A-LYZER G2 dialysis tubing (MWCO, 1000 kDa). In cellular binding and internalization experiments, noncytotoxic FITC-dextran (MW, 10 kD)–encapsulating liposome (DCL-FITC) was prepared and tested to replace the cytotoxic DCL-Dox. The preparation process was similar to that of DCL-Dox except that 1 ml of lipid solution was added to a 9-ml of FITC-dextran solution (1 mg/ml). DiR-labeled DCL (DCL-DiR) was also prepared for in vivo NIR imaging experiments by adding 1 mol % (mole percent) DiR to the lipid composition to prepare the dry lipid film while maintaining the rest steps as the same.

The density of ICAM1 and EGFR antibodies conjugated on liposomes was quantified via microbead assay as described previously (*11*). Liposomes cannot be detected by flow cytometry because of their size, and therefore, 2-μm borosilicate beads were encapsulated within DOPC:DSPE-PEG-COOH (95:5, mol/mol) liposomes by agitating small unilamellar liposomes with microbeads in PBS for 6 hours. Microbeads were rinsed three times in PBS via suspension-spin cycles to separate free liposomes. Conjugation of FITC-ICAM1 antibody, PE-EGFR antibody, or PE-IgG (nonspecific binding) to microbead encapsulating liposomes was performed using EDC/NHS chemistry. Surface densities and ratios of ICAM1 and EGFR antibody conjugated to each microbead were determined with reference to Quantum Simply Cellular microbeads, which have defined numbers of antibody-binding sites per bead. The size and surface charge of DCLs and control liposomes were determined by dynamic light scattering on a ZetaPALS analyzer (Brookhaven Instruments, Holtsville, NY, USA). Liposome samples were diluted in PBS (pH 7.4) at a lipid concentration of 100 μM at 25°C and measured with using a scattering angle of 90° and unimodal analysis. The zeta potential was calculated from the electrophoretic mobility by means of the Hemholtz-Smoluchowski relation. All measurements were performed in triplicate. The morphology of DCLs was determined by transmission electron microscope (TEM) imaging on a JEOL 2100 TEM (JEOL, Peabody, MA, USA). Freshly prepared liposome samples (5 μl, 100 μM in deionized water) were dropped onto a 300-mesh carbon-coated copper grid (Ted Pella, Inc., Redding, CA, USA) and dried. All images were acquired at an accelerating voltage of 100 kV.

The Dox-encapsulating efficiency in DCL-Dox and control liposomes was determined using a fluorescent assay. A Dox calibration curve was generated from a series of serially diluted free Dox standard solutions, and appropriate backgrounds were measured on a SpectraMaxPlus 384 UV-Visible spectrophotometer (excitation, 485 nm; emission, 590 nm; Molecular Devices, San Jose, CA, USA). A 20-μl liposome sample was added to 980 μl of 0.5% Triton X-100 in a microcentrifuge tube and vortexed for 1 min. The microcentrifuge tube was transferred to a 37°C incubator for 1 hour. Triton X-100 is a surfactant that lyses liposomes. Then, 200 μl of the Dox containing Triton X-100 solution was added to at least three wells for each sample of a flat bottom 96-well cell culture plate and measured for fluorescence. The 0.5% Triton X-100 solution without any liposome sample was used as a blank control. The Dox encapsulation efficiency is calculated from the following formula: encapsulated Dox concentration/initial Dox concentration × 100.

### Cellular binding and internalization assay

Quantitative analysis of liposome binding to TNBC cells was studied by flow cytometry analysis. Cells (10^6^) were placed in each well of a six-well cell culture plate and incubated for 4 hours at 37°C with IgG-FITC-LP, ICAM-FITC-LP, EGFR-FITC-LP, DCL-FITC_4.2/1, DCL-FITC_1.5/1,DCL-FITC_1/1, ICAM-FITC-LP/EGFR-FITC-LP mixture (4.2/1 ratio), ICAM-FITC-LP/EGFR-FITC-LP mixture (1.5/1 ratio), and ICAM-FITC-LP/EGFR-FITC-LP mixture (1/1 ratio) at a final concentration of 1 μM lipids per 10^6^ cells. All liposome-treated cells were washed with PBS, harvested using a 0.25% trypsin/2.6 mM EDTA solution, and washed three times with PBS (pH 7.4). Binding data were acquired using a BD FACSCalibur flow cytometer and analyzed using FlowJo software. Cellular binding and uptake of DCLs were calculated by dividing the mean fluorescence intensity of DCL-FITC–treated cells by that of the IgG-FITC-LP–treated cells.

The internalization ratio of DCL was evaluated using trypan blue quenching assay as previously reported (*55*, *56*). Briefly, 10^6^ liposome-treated cells collected for flow cytometric analysis were equally divided into two parts. One part was directly used for flow cytometric measurement, and the fluorescence intensity of liposome-treated cells was defined as the total fluorescence including both extracellular and internalized DCLs. The other part was incubated with trypan blue solution (1 mg/ml) for 30 min to quench extracellular fluorescence and washed with PBS. The fluorescence intensity of trypan blue–quenched cells was defined as the internalized fluorescence. The internalization ratio was calculated by dividing internalized fluorescence with total cell fluorescence times 100.

### Cytotoxicity assay

The cytotoxicity of DCL-Dox was evaluated using a cell viability assay. Briefly, 10^4^ cells (MDA-MB-231 and MDA-MB-436) were seeded in each well of a 96-well plate and incubated for 24 hours. Then, cells were treated with PBS, free Dox, IgG-Dox-LP, ICAM-Dox-LP, EGFR-Dox-LP, and DCL-Dox at Dox concentrations ranging from 0 to 50 μg/ml for 6 hours. Cells were rinsed twice with PBS and grown for 48 hours. Cell viability was determined using a Dojindo cell counting kit according to the protocol provided by the manufacturer.

### Cell proliferation assay

Five thousand cells were seeded in each well of a 96-well plate and grown overnight. Then, cells were incubated with PBS, IgG-LP, ICAM-LP, EGFR-LP, and DCL at the final liposome concentration of 1 μM lipids per 10^6^ cells for 48 hours. Cell proliferation was analyzed using a Dojindo cell counting kit.

### Cell invasion assay

One million cells seeded in six-well plate were treated with PBS, IgG-LP, ICAM-LP, EGFR-LP, and DCL at the final liposome concentration of 1 μM lipids per 10^6^ cells for 24 hours and then reseeded onto 24-well Corning BioCoat Matrigel Invasion Chamber system with permeable support polycarbonate membrane (with 8 μm pore size) at a cell density of 10^5^ cells per well. DMEM without FBS and DMEM with 10% FBS were added to the upper and lower wells, respectively. Cells were allowed to invade for 20 hours. Cells on the reverse side of transwell membrane facing the lower chamber after transmigrating through the 8-μm pores of transwell membrane were stained with Diff-Quik Stain Set. Four fields were counted for each sample.

### Orthotopic tumor model and treatments

Animal studies were performed according to the protocols approved by the Institutional Animal Care and Use Committees of Boston Children’s Hospital and The City College of New York. Breast tumors were orthotopically implanted by injecting 5 × 10^6^ MDA-MB-231-Luc cells into the fourth right mammary fat pad of female nude mice (Charles River, Wilmington, MA, USA). Tumor-bearing mice were randomized into various treatment groups (*n* = 7 to 10 per group). For in vivo NIR fluorescent imaging experiments, tumors were allowed to develop for 2 to 3 weeks until they were at least 200 mm^3^ in volume. In vivo NIR fluorescent imaging was performed on the tumor-bearing mice that were intravenously injected with liposomes at a dosage of 20 mg lipids/kg mouse weight using tail vein injection. At 4, 24, and 48 hours after the injection, in vivo NIR fluorescence imaging was performed using an IVIS Lumina II system (Caliper, Hopkinton, MA, USA). At 48 hours after injection, mice were euthanized, and ex vivo NIR fluorescence intensity of various organs (brain, heart, liver, lung, kidney, and spleen) and excised tumors was measured using IVIS Lumina II.

For therapeutic efficacy experiments, MDA-MB-231-Luc tumors were allowed to develop for 1 to 2 weeks until they reached 100 mm^3^ in volume. Mice were randomly divided into different groups and were treated with DCL-Dox or controls at a Dox dose of 2.5 mg/kg per half week. All treatments were performed intravenously via retro-orbital injection in 50-μl volume. Tumor growth was monitored weekly using caliper. Twenty-four days after treatment, orthotopic tumors were excised to measure their mass, and various organs (brain, heart, liver, lung, kidney, and spleen) were collected and analyzed for metastasis using IVIS Lumina II.

### Lung metastasis model and treatments

One million MDA-MB-231-Luc cells in 100 μl of PBS were injected to the lateral tail vein of female nude mice to allow the formation of lung metastasis. At 24 hours after injection, in vivo bioluminescence imaging was performed to confirm the localization of MDA-MB-231-Luc cells in mouse lungs using an IVIS Lumina II system. Then, mice were randomized into six groups (*n* = 8 per group) and received treatments with PBS (sham), free Dox, IgG-Dox-LP, ICAM-Dox-LP, EGFR-Dox-LP, or DCL-Dox_4.2/1 (2.5 mg/kg per dosage, twice a week) for 21 days. All injections for treatments were performed intravenously via retro-orbital injection in 50-μl volume. Lung metastasis of MDA-MB-231-Luc was monitored by weekly in vivo bioluminescence imaging for up to 124 days. Mice were euthanized, and organs were excised to estimate the metastatic burden. In dosage-dependent experiments, four dosages of DCL-Dox_4.2/1 [PBS (sham); 0.625, 1.25, and 2.5 mg/kg] were tested in mice with lung metastasis using the same experimental protocol.

Chronic liver and renal toxicities of DCL-Dox were evaluated by measuring AST, ALT, creatinine, and BUN levels in mouse serum after treatment. At day 74 of dosage-dependent experiments, mice were euthanized with CO_2_, and 500 μl of whole blood was collected via cardiac puncturing. Mouse blood was transferred to a BD Vacutainer and incubated for 20 min at RT to allow clotting. Then, serum was collected after centrifuging at 2000*g* for 10 min in a refrigerated centrifuge. Serum levels of ALT, AST, creatinine, and BUN were determined using their activity assay kits purchased from Sigma-Aldrich (St. Louis, MO, USA) with provided protocols.

### Statistical analysis

All of the experimental data were obtained in triplicate and are presented as means ± SD unless otherwise mentioned. One- and two-way analysis of variance (ANOVA) with Bonferroni post hoc tests were used to analyze statistical variance when making multiple comparisons. Log-rank test was used to analyze statistical variance in Kaplan-Meier survival analysis. All statistical analysis was performed using OriginPro 8 software.

## Supplementary Material

http://advances.sciencemag.org/cgi/content/full/5/3/eaav5010/DC1

Download PDF

## SUPPLEMENTARY MATERIALS

Supplementary material for this article is available at http://advances.sciencemag.org/cgi/content/full/5/3/eaav5010/DC1 Fig. S1. Surface protein expression of 68 cancer targets in three human TNBC cell lines and non-neoplastic MCF10A cells. Fig. S2. Morphological characterization of DCL. Table S1. List of cell membrane proteins. Table S2. ICAM1 and EGFR surface density and ratio on human TNBC cells. Table S3. Dynamic light scattering characterization of DCL-Dox and controls. Table S4. Theoretical and experimental densities of ICAM1 and EGFR antibodies on DCL surfaces.
