# Supplementary material for: Dual complementary liposomes inhibit triple-negative breast tumor progression and metastasis
Source: Sci Adv. 2019 Mar 20;5(3):eaav5010. doi: 10.1126/sciadv.aav5010 (PMC6426465; doi:10.1126/sciadv.aav5010)
Supplement: http://advances.sciencemag.org/cgi/content/full/5/3/eaav5010/DC1 [file supp_5_3_eaav5010__index.html]

Science Advances | Science Advances

## Supplementary Materials

**This PDF file includes:**

- Fig. S1. Surface protein expression of 68 cancer targets in three human TNBC cell lines and non-neoplastic MCF10A cells.
- Fig. S2. Morphological characterization of DCL.
- Table S1. List of cell membrane proteins.
- Table S2. ICAM1 and EGFR surface density and ratio on human TNBC cells.
- Table S3. Dynamic light scattering characterization of DCL-Dox and controls.
- Table S4. Theoretical and experimental densities of ICAM1 and EGFR antibodies on DCL surfaces.

Download PDF

**Files in this Data Supplement:**

- Adobe PDF - aav5010\_SM.pdf
