# Supplementary material for: Dual complementary liposomes inhibit triple-negative breast tumor progression and metastasis
Source: Sci Adv. 2019 Mar 20;5(3):eaav5010. doi: 10.1126/sciadv.aav5010 (PMC6426465; doi:10.1126/sciadv.aav5010)
Supplement: Download PDF [file aav5010_SM.pdf]

Science Advances
AAAS

advances.sciencemag.org/cgi/content/full/5/3/eaav5010/DC1

Supplementary Materials for

Dual complementary liposomes inhibit triple-negative breast tumor progression and metastasis

Peng Guo, Jiang Yang, Daxing Liu, Lan Huang, Gillian Fell, Jing Huang, Marsha A. Moses*, Debra T. Auguste*

*Corresponding author. Email: d.auguste@northeastern.edu (D.T.A.); marsha.moses@childrens.harvard.edu (M.A.M.)

Published 20 March 2019, *Sci. Adv.* **5**, eaav5010 (2019)
DOI: 10.1126/sciadv.aav5010

This PDF file includes:

Fig. S1. Surface protein expression of 68 cancer targets in three human TNBC cell lines and non-neoplastic MCF10A cells.
Fig. S2. Morphological characterization of DCL.
Table S1. List of cell membrane proteins.
Table S2. ICAM1 and EGFR surface density and ratio on human TNBC cells.
Table S3. Dynamic light scattering characterization of DCL-Dox and controls.
Table S4. Theoretical and experimental densities of ICAM1 and EGFR antibodies on DCL surfaces.

Supplementary Materials

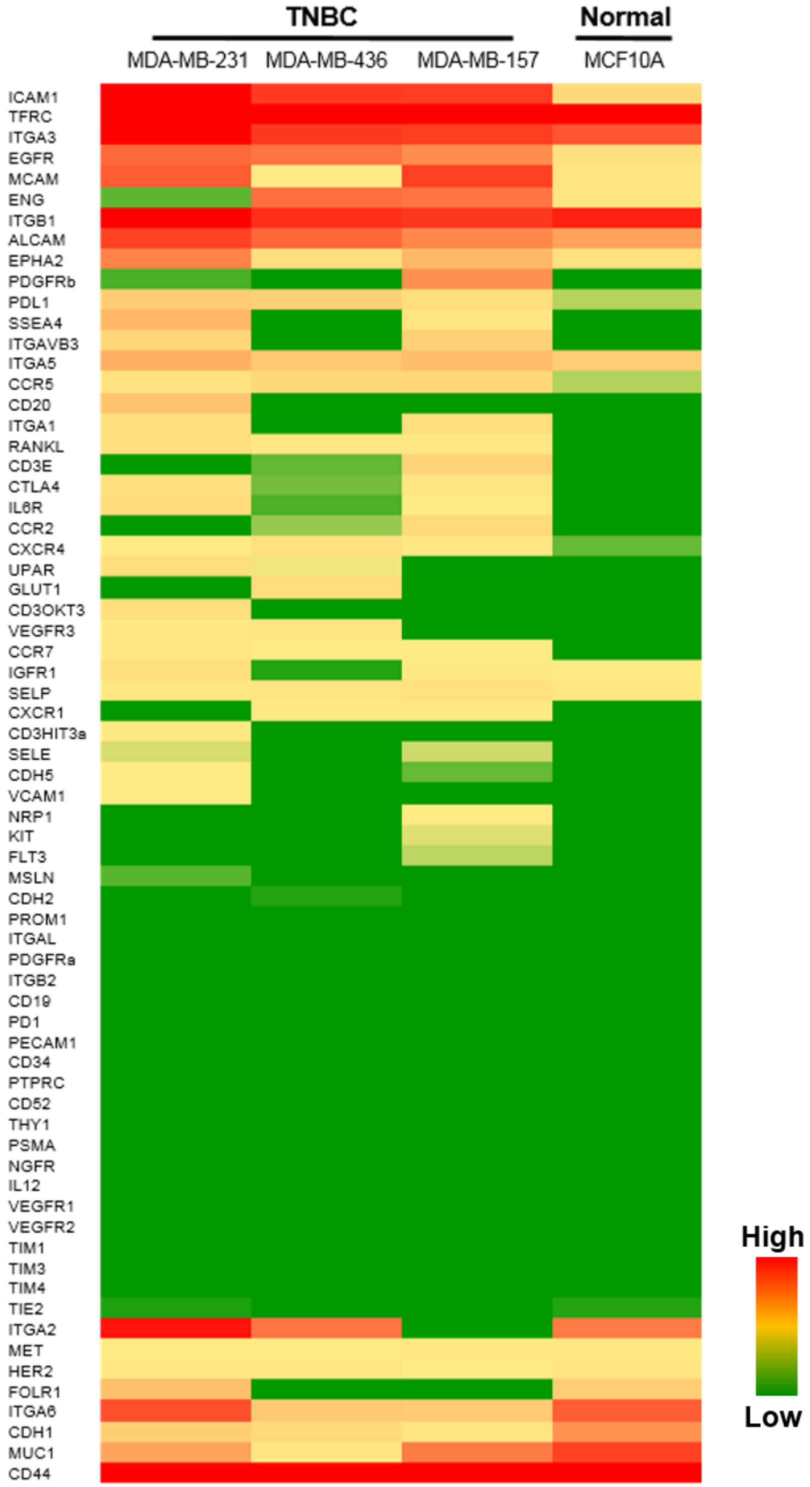

Fig. S1. Surface protein expression of 68 cancer targets in three human TNBC cell lines and non-neoplastic MCF10A cells. Red and green bars represent maximum and minimum expression respectively.

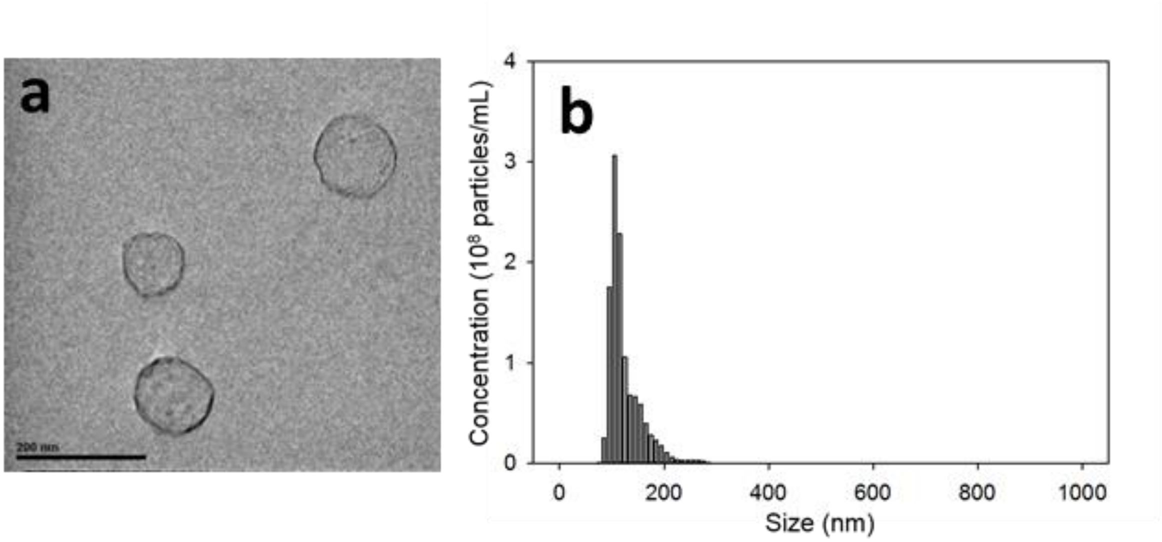

Fig. S2. Morphological characterization of DCL. (**a**) Transmission electron microscopy image of dual complementary liposomes (DCL without payload). Scale bar represents 200 nm. (**b**) Hydrodynamic radius of DCLs as analyzed by dynamic light scattering measurement.

Table S1. List of cell membrane proteins.

Name	Description
**ALCAM**	Activated leukocyte cell adhesion molecule
**CCR2**	Chemokine (C-C motif) receptor 2
**CCR5**	Chemokine (C-C motif) receptor 5
**CCR7**	Chemokine (C-C motif) receptor 7
**CD19**	CD19 molecule
**CD20**	CD20 molecule
**CD34**	CD34 molecule
**CD3E**	CD3e molecule, epsilon
**CD3HIT3a**	CD3 molecule, HIT3a
**CD3OKT3**	CD3 molecule, OKT3
**CD44**	CD44 molecule

**CD52**	CD52 molecule
**CDH1**	Cadherin 1, type 1, E-cadherin (epithelial)
**CDH2**	Cadherin 2, type 1, N-cadherin
**CDH5**	Cadherin 5, type 2 (vascular endothelium)
**CTLA4**	Cytotoxic T-lymphocyte-associated protein 4
**CXCR1**	Chemokine (C-X-C motif) receptor 1
**CXCR4**	Chemokine (C-X-C motif) receptor 4
**EGFR**	Epidermal growth factor receptor
**ENG**	Endoglin
**EPHA2**	EPH receptor A2
**FLT3**	Fms-related tyrosine kinase 3
**FOLR1**	Folate receptor 1
**GLUT1**	Glucose transporter 1
**HER2**	human epidermal growth factor receptor 2
**ICAM1**	Intercellular adhesion molecule 1
**IGFR1**	Insulin-like growth factor 1 receptor
**IL12**	Interleukin 12
**IL6R**	Interleukin 6 receptor
**ITGA1**	Integrin, alpha 1
**ITGA2**	Integrin, alpha 2
**ITGA3**	Integrin, alpha 3
**ITGA5**	Integrin, alpha 5
**ITGA6**	Integrin, alpha 6
**ITGAL**	Integrin, alpha L
**ITGAVB3**	Integrin alpha V beta 3

**ITGB1**	Integrin, beta 1
**ITGB2**	Integrin, beta 2
**KIT**	Mast/stem cell growth factor receptor
**MCAM**	Melanoma cell adhesion molecule
**MET**	MET proto-oncogene, receptor tyrosine kinase
**MSLN**	Mesothelin
**MUC1**	Mucin 1, cell surface associated
**NGFR**	Nerve Growth Factor Receptor
**NRP1**	Neuropilin 1
**PD1**	Programmed cell death protein 1
**PDGFRA**	Platelet-derived growth factor receptor, alpha polypeptide
**PDGFRB**	Platelet-derived growth factor receptor, beta polypeptide
**PDL1**	Programmed death-ligand 1
**PECAM1**	Platelet/endothelial cell adhesion molecule 1
**PROM1**	Prominin 1
**PSMA**	Prostate-specific membrane antigen
**PTPRC**	Protein tyrosine phosphatase, receptor type, C
**RANKL**	Receptor activator of nuclear factor kappa-B ligand
**SELE**	Selectin E
**SELP**	Selectin P
**SSEA4**	Stage specific embryonic antigen 4
**TFRC**	Transferrin receptor
**THY1**	Thy-1 cell surface antigen
**TIE2**	TEK tyrosine kinase, endothelial
**TIM1**	T-cell immunoglobulin and mucin domain 1

**TIM3**	T-cell immunoglobulin and mucin-domain 3
**TIM4**	T-cell immunoglobulin and mucin-domain 4
**UPAR**	Plasminogen activator, urokinase receptor
**VCAM1**	Vascular cell adhesion molecule 1
**VEGFR1**	Vascular endothelial growth factor receptor 1
**VEGFR2**	Vascular endothelial growth factor receptor 2
**VEGFR3**	Vascular endothelial growth factor receptor 3

Table S2. ICAM1 and EGFR surface density and ratio on human TNBC cells.

Cell line	ICAM1 surface density (molecules/cell)	EGFR surface density (molecules/cell)	Total surface density (molecules/cell)	ICAM1/EGFR Protein Ratio
MDA-MB-231	2,350,000 ± 25,000	559,000 ± 1,200	2,909,000	4.2:1
MDA-MB-436	756,000 ± 7,600	514,000 ± 2,200	1,270,000	1.5:1
MDA-MB-157	751,000 ± 4,400	406,000 ± 8,900	1,157,000	1.8:1
MCF10A	93,000 ± 2,300	61,200 ± 740	154,200	1.5:1

Table S3. Dynamic light scattering characterization of DCL-Dox and controls.

Sample	Size (nm)	PDI	Zeta-potential (mV)	Dox Encapsulation Efficiency (%)
IgG-Dox-LP	128 ± 32	0.050	-10.8 ± 0.7	98.1 ± 2.2
ICAM1-Dox-LP	123 ± 21	0.022	-8.2 ± 1.9	97.8 ± 0.7
EGFR-Dox-LP	125 ± 25	0.026	-8.0 ± 0.6	97.6 ± 2.3
DCL-Dox_4.2/1	132 ± 20	0.015	-6.3 ± 1.6	98.6 ± 2.2
DCL-Dox_1.5/1	133 ± 26	0.022	-5.6 ± 0.7	98.5 ± 0.4
DCL-Dox_1/1	132 ± 13	0.009	-6.2 ± 0.9	97.9 ± 2.9

Table S4. Theoretical and experimental densities of ICAM1 and EGFR antibodies on DCL surfaces.

Sample	ICAM1/EGFR Antibody Ratio (Theoretical)	ICAM1 Density (molecules/μm^2)	EGFR Density (molecules/μm^2)	IgG Density (molecules/μm^2)	Total Antibody Density (molecules/μm^2)	ICAM1/EGFR Antibody Ratio (Experimental)
IgG-Dox-LP	NA	0	0	4,236 ± 180	4,236 ± 180	NA
ICAM1-Dox-LP	1:0	4,527 ± 316	0	0	4,527 ± 316	1:0
EGFR-Dox-LP	0:1	0	4,455 ± 43	0	4,455 ± 43	0:1
DCL-Dox_4.2/1	4.2:1	3,647 ± 93	925 ± 88	0	4,572 ± 181	4.0:1
DCL-Dox_1.5/1	1.5:1	2,609 ± 42	2,230 ± 43	0	4,840 ± 6	1.2:1
DCL-Dox_1/1	1:1	2,406 ± 88	2,364 ± 132	0	4,770 ± 173	1.02:1
